# Targeting DNA Damage Response and Immune Crosstalk in Cancer: Mechanistic Insights and Therapeutic Opportunities

**DOI:** 10.3390/ijms262311271

**Published:** 2025-11-21

**Authors:** Lavinia Marcut, Roxana Daniela Brata, Alina Cristina Barb, Alexia Manole, Dan Gabriel Stef, Cristina Stefania Dumitru, Flavia Zara, Raul Patrascu

**Affiliations:** 1 Department of Surgical Sciences, Faculty of Medicine and Pharmacy, University of Oradea, 410073 Oradea, Romania; lmarcut@uoradea.ro; 2 Department of Medical Disciplines, Faculty of Medicine and Pharmacy, University of Oradea, 410073 Oradea, Romania; brata.roxanadaniela@didactic.uoradea.ro; 3 Department II of Microscopic Morphology, Discipline of Histology, Victor Babes University of Medicine and Pharmacy Timisoara, E. Murgu Square, No. 2, 300041 Timisoara, Romania; toma.alina@umft.ro (A.C.B.); flavia.zara@umft.ro (F.Z.); 4 Faculty of Medicine and Pharmacy, University of Oradea, 410087 Oradea, Romania; manole.alexia@student.uoradea.ro; 5 Department IX, Discipline of Surgical Semiology I, Victor Babes University of Medicine and Pharmacy Timisoara, E. Murgu Square, No. 2, 300041 Timisoara, Romania; dan.stef@umft.ro; 6 Department of Functional Sciences, Victor Babes University of Medicine and Pharmacy, 300041 Timisoara, Romania; patrascu.raul@umft.ro

**Keywords:** DNA damage response, immune crosstalk, cGAS–STING pathway, PARP inhibitors, ATR inhibitors, tumor microenvironment, genomic instability, precision oncology

## Abstract

Cancer progression and therapeutic resistance are driven by complex molecular interactions between genomic instability and immune modulation. Defects in the DNA damage response (DDR) not only promote tumor heterogeneity but also shape the tumor immune landscape through the generation of neoantigens, activation of the cGAS–STING pathway, and modulation of immune checkpoints. This review provides an integrative overview of the molecular mechanisms linking DDR dysfunction to immune crosstalk, emphasizing how these processes influence tumor evolution and response to therapy. We discuss emerging therapeutic strategies that exploit DDR–immune interactions, including PARP and ATR inhibitors, synthetic lethality approaches, and combination regimens with immune checkpoint blockade. Understanding the bidirectional connection between DNA repair pathways and immune signaling unveils new translational opportunities for precision oncology and offers a framework for developing combinatorial therapies capable of overcoming resistance and improving long-term cancer control.

## 1. Introduction

Cancer is a multifactorial disease driven by the accumulation of genetic and epigenetic alterations that disrupt cellular homeostasis and promote uncontrolled proliferation, invasion, and metastasis [1]. Among the established hallmarks of cancer, genomic instability stands as a central enabling factor that sustains tumor heterogeneity and evolution. The DNA damage response (DDR) represents a complex network of signaling pathways responsible for detecting and repairing DNA lesions, thus maintaining genomic integrity. When DDR pathways are compromised—through germline mutations, somatic alterations, or epigenetic silencing—cells accumulate mutations that accelerate oncogenesis and modulate therapeutic responses [2,3].

In parallel, the immune system exerts constant surveillance against malignant transformation by recognizing and eliminating aberrant cells. Nonetheless, tumors evolve strategies to evade immune detection, such as downregulating antigen presentation, secreting immunosuppressive cytokines, and remodeling the tumor microenvironment toward tolerance [4]. Recent findings have revealed a profound interconnection between DDR defects and immune modulation. DNA damage enhances tumor mutational burden and neoantigen formation, which can increase tumor immunogenicity. Moreover, cytosolic DNA fragments arising from defective repair activate the cGAS–STING pathway, inducing type I interferon signaling and bridging innate and adaptive immune responses [5,6,7]. However, persistent activation of this axis may lead to chronic inflammation, immune exhaustion, and tumor progression [8].

Understanding the molecular interplay between DDR signaling and immune crosstalk holds profound implications for precision oncology. Therapeutic agents that target DDR—such as PARP, ATR, and DNA-PK inhibitors—have demonstrated clinical efficacy, particularly in tumors with homologous recombination repair deficiencies. Furthermore, the combination of DDR-targeting drugs with immune checkpoint inhibitors (ICIs) has emerged as a promising strategy to enhance antitumor immunity and overcome resistance to monotherapies [9,10,11]. Despite these advances, the molecular determinants governing DDR–immune interactions remain incompletely elucidated and context-dependent.

This review aims to synthesize the current understanding of the mechanistic connections between DNA damage response pathways and immune modulation in cancer. We discuss how DDR dysfunction alters the tumor immune landscape, the therapeutic opportunities emerging from this bidirectional interaction, and the translational challenges that remain. By integrating molecular, preclinical, and clinical evidence, this review underscores the therapeutic promise of targeting DDR–immune crosstalk to improve outcomes and guide the next generation of combination therapies in oncology.

## 2. DNA Damage Response Pathways and Their Role in Cancer

The integrity of the genome is continuously threatened by both endogenous and exogenous sources of DNA damage, including reactive oxygen species generated during cellular metabolism, replication stress, ultraviolet (UV) radiation, and exposure to genotoxic chemicals. To safeguard genomic stability, eukaryotic cells have developed an intricate surveillance and repair network collectively referred to as the DNA damage response (DDR). This coordinated system governs lesion detection, activation of checkpoint signaling cascades, recruitment of repair complexes, and, when necessary, the induction of apoptosis or senescence to prevent the transmission of genetic errors to progeny cells. When DDR pathways are disrupted—through mutations, deletions, or epigenetic silencing—cells accumulate genomic alterations that fuel oncogenic transformation, chromosomal instability, and resistance to anticancer therapies [2].

The DDR encompasses multiple, highly conserved repair mechanisms, each specialized for distinct types of DNA lesions. Base excision repair (BER) corrects small base modifications caused by oxidation or alkylation, while nucleotide excision repair (NER) removes bulky adducts and UV-induced thymine dimers. Mismatch repair (MMR) resolves replication errors, ensuring fidelity during DNA synthesis. In contrast, DNA double-strand breaks—among the most cytotoxic forms of damage—are repaired by homologous recombination (HR) or non-homologous end joining (NHEJ), depending on cell-cycle context [12,13].

Key regulatory kinases—ataxia telangiectasia mutated (ATM) and Rad3-related (ATR), and DNA-dependent protein kinase catalytic subunit (DNA-PKcs)—act as central nodes in DDR signaling. Upon sensing DNA lesions, ATM and ATR activate distinct downstream branches: ATM primarily phosphorylates CHK2 and p53 to enforce G1 and G2 checkpoint arrest, whereas ATR signals mainly through CHK1 to stabilize replication forks and suppress premature S–M transition. Both kinases also modulate key repair factors such as BRCA1, RAD51, and XRCC4, thereby linking checkpoint activation with pathway-specific repair execution [14,15].

Defects in DDR components not only initiate tumorigenesis but also create vulnerabilities that can be therapeutically exploited. The synthetic lethality observed between BRCA1/2 mutations and poly(ADP-ribose) polymerase (PARP) inhibition exemplifies how DDR-targeted therapies can selectively kill cancer cells with homologous recombination deficiencies while sparing normal tissues [16,17].

Collectively, these findings underscore that DDR pathways function not merely as genome caretakers but as dynamic regulators of tumor evolution and therapeutic response. Understanding their molecular interconnections is crucial for identifying predictive biomarkers, guiding treatment selection, and designing rational combination strategies in precision oncology.

### 2.1. Classification of DNA Repair Mechanisms

To maintain genomic integrity under persistent genotoxic stress, eukaryotic cells deploy distinct DNA repair pathways that operate in a lesion- and cell-cycle-specific manner, coordinating damage detection, excision or end processing, gap filling, and ligation. These pathways are tightly regulated to prevent mutational fixation and chromosomal instability that fuel oncogenesis [13].

Base Excision Repair (BER). BER corrects small, non-helix-distorting lesions produced by oxidation, deamination, or alkylation. Lesion-specific DNA glycosylases initiate repair by generating an abasic (AP) site, which is incised by APE1; DNA polymerase β then inserts the correct nucleotide, and XRCC1–LIG3 seals the nick. Emerging oncologic data link BER gene defects (e.g., MUTYH, OGG1) with characteristic mutational signatures and therapeutic vulnerabilities. Recent reviews also highlight BER’s interface with redox homeostasis and chemotherapy response [18].

Nucleotide Excision Repair (NER). NER removes bulky, helix-distorting lesions such as UV-induced cyclobutane pyrimidine dimers and chemical adducts via two sub-branches: global-genome NER (GG-NER) and transcription-coupled NER (TC-NER). In GG-NER, XPC–HR23B senses lesions; in TC-NER, RNA Pol II stalling recruits CSA/CSB. TFIIH (XPB/XPD helicases) unwinds DNA; XPF–ERCC1 and XPG execute dual incision; gap filling and ligation restore the duplex. High-resolution structural and single-molecule studies from the last decade have refined our understanding of TFIIH activation and XPG’s incision licensing [19,20,21,22]. Recent cryo-EM reconstructions revealed that TFIIH undergoes a transition from a closed to an open translocase conformation, enabling XPB-driven DNA opening and positioning XPD for lesion verification. Single-molecule FRET studies further demonstrated that XPG loading is temporally coupled to TFIIH’s helicase activity, with XPG docking onto the pre-incision bubble only after TFIIH achieves a stable unwound state. These insights clarify how structural gating by TFIIH licenses the 3′ incision by XPG and ensures incision fidelity during NER [23,24].

Beyond its mechanistic function, NER deficiency carries clear biological and pathological consequences. Germline defects in core NER components such as XPA, XPC, ERCC2, or ERCC3 underlie xeroderma pigmentosum and trichothiodystrophy, disorders characterized by extreme UV sensitivity and markedly increased skin cancer risk due to impaired removal of bulky adducts [25]. Somatic ERCC2 and ERCC1 alterations also influence therapeutic response, particularly to platinum-based chemotherapy, where reduced NER capacity enhances cisplatin sensitivity but also predisposes to treatment-related toxicities. Emerging evidence further shows that NER loss increases mutational burden and cytosolic DNA accumulation, promoting type I interferon signaling and enhancing tumor immunogenicity—features with relevance for response to immune checkpoint blockade [26].

Mismatch Repair (MMR). MMR safeguards replication fidelity by resolving base–base mismatches and insertion–deletion loops. MutSα (MSH2–MSH6) or MutSβ (MSH2–MSH3) recognizes errors; MutLα (MLH1–PMS2) coordinates excision and resynthesis. Loss of MMR produces microsatellite instability (MSI), a hallmark of Lynch-spectrum tumors and a predictive biomarker for immune checkpoint blockade—now codified in testing guidelines. Contemporary clinical meta-analyses broaden MSI/dMMR relevance beyond colorectal cancer (e.g., ovarian and endometrial) [27,28,29].

Double-Strand Break (DSB) Repair. DSBs are the most cytotoxic lesions; their accurate resolution prevents translocations and aneuploidy. Two principal pathways operate in a cell-cycle-dependent manner: Homologous Recombination Repair (HRR) and Non-Homologous End Joining (NHEJ).

HRR operates exclusively during the S and G2 phases, when the sister chromatid is available as an accurate repair template. BRCA1/2 orchestrate end resection and RAD51 filament formation, enabling high-fidelity strand invasion. Clinically, HRR defects extend beyond BRCA1/2, encompassing PALB2, BARD1, RAD51 paralogs and Fanconi anemia genes; RAD51 foci assays are emerging as functional biomarkers to guide PARP inhibitor use and combination strategies [30,31,32].

NHEJ functions throughout the cell cycle and is intrinsically error-prone. Ku70/Ku80 recognizes DSB ends and recruits DNA-PKcs; synapsis is stabilized as limited end processing prepares termini for ligation by LIG4–XRCC4–XLF. Recent single-molecule and cryo-EM studies have revealed that DNA-PK–bound DNA ends can adopt extended and compact synaptic conformations, with autophosphorylation-driven conformational opening being essential for end-processing and ligation. Disruption of this regulated synapsis—either by impairing DNA-PKcs autophosphorylation or destabilizing the long-range synaptic complex—sensitizes cells to ionizing radiation and underlies the mechanism by which DNA-PK–targeted inhibitors enhance radiosensitization [33,34,35].

Synthesis and clinical note. The division of labor among BER, NER, MMR, HRR, and NHEJ creates a multilayered defense that preserves genome stability while shaping drug response. Selective pathway loss in cancer constitutes both a driver of tumorigenesis and a therapeutic vulnerability—supporting synthetic-lethal strategies and biomarker-driven combinations [36,37].

### 2.2. DDR Signaling and Checkpoint Activation

The DNA damage response (DDR) is orchestrated by a triad of phosphatidylinositol 3-kinase-related kinases (PIKKs): ATM (ataxia-telangiectasia mutated), ATR (ATM and Rad3 related) and DNA-PKcs (DNA-dependent protein kinase catalytic subunit). These kinases act at the apex of DDR signaling, sensing distinct types of DNA lesions and initiating checkpoint activation, repair recruitment and cell-cycle control. For example, structural analyses demonstrate that ATM, ATR, and DNA-PKcs share the conserved PIKK catalytic architecture, including the C-terminal FAT, kinase, and FATC domains that define substrate recognition and catalytic regulation. Despite this shared core, each kinase is activated by distinct lesion-specific assemblies: ATM by the MRN complex at double-strand breaks, ATR by RPA-coated single-stranded DNA via ATRIP, and DNA-PKcs by KU70/80 bound to DNA ends [38,39]. ATM is activated primarily in response to double-strand breaks (DSBs) via recruitment by the MRN (MRE11–RAD50–NBS1) complex, and upon activation it phosphorylates a broad spectrum of substrates such as CHK2 and p53, activating the G1 and G2 checkpoints and inducing cell-cycle arrest to allow repair to proceed [40,41]. ATR is activated when replication stress generates stretches of RPA-coated single-stranded DNA, which recruit the ATR–ATRIP complex to stalled forks. It signals mainly through CHK1 to stabilize stalled replication forks, inhibit origin firing, and prevent premature mitotic entry, thereby maintaining replication integrity [41,42].

During canonical NHEJ, the KU70/80 heterodimer is the primary sensor of DNA double-strand breaks, rapidly encircling the DNA ends and subsequently recruiting DNA-PKcs to form the active DNA-PK holoenzyme. End detection by KU70/80 precedes the conformational activation of DNA-PKcs, which undergoes autophosphorylation to orchestrate end-processing and ligation. Beyond its structural role in NHEJ, DNA-PKcs facilitates checkpoint recovery by promoting cell-cycle restart after damage resolution and exhibits functional crosstalk with ATM and ATR through shared substrates such as H2AX, thereby integrating end-joining repair with broader DDR signaling networks [39,42]. Beyond the activation of ATM and ATR, checkpoint enforcement involves a defined cascade of downstream effectors that halt cell-cycle progression [43]. In the G1 checkpoint, ATM-mediated phosphorylation of CHK2 and p53 stabilizes the p53–p21 axis, leading to inhibition of CDK2–cyclin E and preventing the G1/S transition. During the intra-S checkpoint, ATR-driven CHK1 activation suppresses CDC25A phosphatase activity, reducing CDK2-cyclin A activity, limiting origin firing, and stabilizing stalled replication forks. The G2/M checkpoint is similarly governed by ATR–CHK1 and ATM–CHK2 signaling, which inhibit CDC25C and maintain CDK1 in an inactive state through persistent tyrosine-15 phosphorylation, thereby preventing premature entry into mitosis. Together, these pathways ensure that Cdk activity is competitively inhibited until DNA repair is complete, linking lesion detection with precise cell-cycle control [44].

Together, these kinases integrate checkpoint control by halting G1/S, intra-S, and G2/M transitions to provide time for repair. Partial or non-lethal defects in checkpoint signaling allow cells with unrepaired lesions to continue proliferating, promoting genomic instability and tumorigenesis. In contrast, complete loss of ATR or CHK1 activity prevents stabilization of stalled replication forks and leads to replication catastrophe, resulting in rapid cell death rather than transformation. Recent studies demonstrate that simultaneous inhibition of ATM, ATR and DNA-PKcs leads to synergistic accumulation of DNA damage and chromosomal aberrations [45].

**Figure 1:**
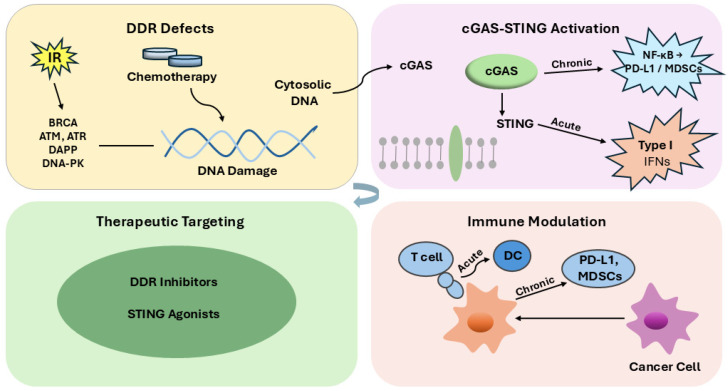
Endogenous or exogenous stressors such as ionizing radiation (IR) and chemotherapy induce DNA lesions that are processed through the five principal repair pathways—base excision repair (BER), nucleotide excision repair (NER), mismatch repair (MMR), homologous recombination repair (HRR), and non-homologous end joining (NHEJ)—each driven by distinct protein complexes (e.g., PARP1/XRCC1 for BER, XPC–ERCC1–XPF/XPG for NER, MLH1–MSH2–PMS2 for MMR, BRCA1/2–RAD51 for HRR, and KU70/80–DNA-PKcs for NHEJ). Upstream kinases such as ATM, ATR, and DNA-PKcs coordinate cell-cycle arrest, repair signaling, or apoptosis. Defective DDR or persistent DNA damage results in the accumulation of cytosolic DNA, which activates cGAS and subsequently STING. The figure distinguishes acute cGAS–STING activation, which triggers IRF3-dependent type I interferon (IFN-I) production and promotes dendritic-cell maturation and cytotoxic T-cell priming, from chronic STING activation, which shifts toward NF-κB signaling, PD-L1 upregulation, and MDSC expansion, collectively generating an immunosuppressive tumor microenvironment. Therapeutic targeting with DDR inhibitors or STING agonists aims to exploit these pathways to enhance antitumor immunity and improve cancer treatment responses.

The major DDR pathways operate through coordinated repair mechanisms that preserve genomic stability and prevent malignant transformation. To provide an integrative overview, Figure 1 schematically illustrates the principal DNA repair pathways, including base excision repair (BER), nucleotide excision repair (NER), mismatch repair (MMR), homologous recombination repair (HRR), and non-homologous end joining (NHEJ). The figure also highlights the central role of the ATM–CHK2 and ATR–CHK1 checkpoint branches, which operate as parallel but interconnected signaling pathways to coordinate cell-cycle arrest and apoptosis following DNA damage.

### 2.3. DDR Deficiency and Tumorigenesis

Defects in the DDR constitute a fundamental driver of oncogenesis by promoting genomic instability, accelerating mutational accumulation, and enabling the emergence of clones that are preferentially selected by microenvironmental, immune-mediated, and therapeutic selective pressures. Large-scale genomic analyses have demonstrated that inactivation of DDR genes—particularly those involved in HRR, MMR, and checkpoint control—correlates with increased tumor mutational burden (TMB) and chromosomal instability (CIN), both of which promote tumor evolution and immune evasion [46,47].

At the molecular level, DDR deficiency acts as a double-edged sword. On the one hand, impaired DNA repair mechanisms such as defective HRR or checkpoint failure drive oncogenic transformation through error-prone repair, replication stress, and chromosomal segregation errors [48]. On the other, these same defects create therapeutic vulnerabilities that can be exploited through synthetic lethality. The prototypical example involves tumors with BRCA1/2 mutations, in which inhibition of poly(ADP-ribose) polymerase (PARP) leads to replication-associated cytotoxicity and cell death. The clinical efficacy of PARP inhibitors such as olaparib has been confirmed across multiple tumor types with HRR deficiency, including breast, ovarian, and prostate cancers [49,50].

Beyond BRCA-driven synthetic lethality, defects in other DDR components also confer targetable susceptibilities. Loss of ATM function disrupts double-strand break signaling and markedly increases replication stress, leading to persistent fork stalling and accumulation of single-stranded DNA. Under these conditions, ATR becomes the dominant survival pathway, stabilizing replication forks and preventing replication-associated collapse. Consequently, ATM-deficient tumors exhibit synthetic-lethal dependence on ATR activity and show pronounced sensitivity to ATR inhibition. This mechanistic relationship has been demonstrated in multiple preclinical studies and corroborated in early-phase clinical trials evaluating ATR inhibitors such as ceralasertib and elimusertib in ATM-mutant malignancies [51,52,53]. In contrast, CHK2 loss produces more complex outcomes: rather than restoring homologous recombination, CHK2 deficiency weakens checkpoint control, allowing proliferation of HR-deficient cells that would otherwise undergo fork collapse. This reduced checkpoint enforcement can attenuate PARP inhibitor sensitivity despite persistent HR defects, underscoring the importance of pathway context when interpreting CHK2 alterations [54,55]. From an evolutionary standpoint, persistent DDR deficiency contributes to increased intratumoral heterogeneity, fostering adaptability under therapeutic stress. Continuous DNA damage and replication stress generate genetically diverse subclones that enhance tumor fitness and drive resistance to cytotoxic, targeted, or immune-based treatments. This dynamic process links DDR dysfunction to therapy-induced evolution and relapse across several cancer types [56,57].

Altogether, DDR deficiency is both a catalyst for malignant transformation and a source of therapeutic opportunity. Understanding the spectrum of repair alterations and their context-dependent vulnerabilities provides a foundation for precision oncology strategies that integrate DDR inhibitors with radiotherapy, chemotherapy, or immunotherapy to improve clinical outcomes.

### 2.4. DDR and Immune Activation

Defects in DDR promote the formation of micronuclei during mitosis, arising from lagging chromosomes caused by unrepaired double-strand breaks; subsequent rupture of the micronuclear envelope releases DNA into the cytosol, leading to robust activation of the cGAS–STING pathway [8]. Micronuclear chromatin is surveilled by cGAS once the micronuclear envelope ruptures, providing a direct route from genome instability to innate immune sensing [58]. Recent high-resolution cryo-EM studies have further shown that cGAMP binding induces STING dimer compaction and higher-order oligomerization, a structural transition that is essential for efficient TBK1 recruitment and downstream IRF3 activation. The cGAS–cGAMP–STING axis is the dominant DNA-sensing pathway in mammalian cells and triggers TBK1–IRF3 signaling to induce type I interferons and inflammatory cytokines [59]. Within cancers, cGAS–STING signaling links chromosomal instability to antitumor immunity but can also be co-opted by tumors, underscoring its context-dependent roles [60]. Type I interferon outputs from cGAS–STING activation support dendritic-cell maturation and cross-priming, enhancing T-cell–mediated tumor immunity [61].

Therapeutically induced DNA damage can amplify these responses: PARP inhibition in BRCA-deficient models activates STING signaling and requires CD8^+^ T-cell recruitment for efficacy [6]. Similarly, ATR inhibition augments the radiation-induced inflammatory tumor microenvironment and increases chemokine/cytokine programs consistent with enhanced immune activation [11]. In BRCA2-deficient settings, chronic DNA damage elevates innate immune gene programs that are further potentiated by PARP inhibitors, reinforcing DDR–immune coupling [10].

Defective DNA repair also increases TMB, broadening the repertoire of neoantigens available for cytotoxic T-lymphocyte recognition and correlating with improved survival following immune checkpoint blockade across multiple histologies [62].

Conversely, sustained or maladapted DDR-linked interferon signaling can drive immune suppression: chronic IFN programs promote PD-L1 upregulation and T-cell exhaustion, mediating adaptive resistance to checkpoint therapy [63,64]. Multiple contemporary analyses further detail how interferon-driven states and tumor-intrinsic programs contribute to primary and acquired resistance to PD-1/PD-L1 blockade. STING pathway activation modulates myeloid compartments; dosing and context determine whether responses favor productive CD8^+^ T-cell immunity or recruitment/expansion of suppressive populations such as MDSCs [65,66]. These dual effects help explain why clinical translation of STING agonists has required optimization of drug design, delivery, and combination strategies [67,68].

Overall, DDR dysfunction shapes a bidirectional interface with the immune system—capable of increasing immunogenicity via cytosolic DNA and neoantigen generation, yet also of driving chronic inflammatory circuits that sustain checkpoint ligand expression and T-cell dysfunction—highlighting the therapeutic opportunity (and necessity) to tune this axis with rational combinations [7].

To summarize the diverse molecular mechanisms described above, Table 1 provides a concise overview of the main DDR pathways, their key molecular mediators, and the corresponding immunologic consequences relevant to cancer biology. This integrative perspective highlights how distinct repair defects—ranging from base excision to double-strand break repair—differentially influence tumor immunogenicity and responsiveness to therapy. The table also indicates representative therapeutic implications, reflecting how the manipulation of DDR components can enhance antitumor immunity or modulate immune evasion mechanisms.

## 3. Intersections Between DNA Damage Response and Immune Signaling Pathways

The interplay between the DNA damage response (DDR) and immune signaling forms a dynamic regulatory network in which chromosomal instability and cytosolic DNA sensing can either stimulate antitumor immunity or be co-opted to promote immune evasion, depending on cellular and microenvironmental context [7]. Defective DDR reshapes the mutational landscape and increases the probability of immunogenic neoantigen formation (higher tumor mutational burden), thereby influencing responses to immune checkpoint blockade across multiple cancers [69].

Mechanistically, cytosolic DNA generated by unrepaired lesions or micronuclei activates the cGAS–STING pathway, linking genomic instability with type I interferon signaling and antigen-presentation programs. These immune-stimulatory outputs enhance responsiveness to immune checkpoint blockade but can also drive adaptive resistance under chronic activation [70,71]. These intersections supply the conceptual and translational basis for rational combinations of DDR-targeted agents (e.g., PARP, ATR, DNA-PK inhibitors) with immunomodulatory approaches, a strategy now supported by state-of-the-art reviews and emerging clinical efforts [72].

### 3.1. Cytosolic DNA Sensing and cGAS–STING Activation

The cGAS–STING pathway acts as the principal sensor–effector module linking genomically unstable tumors to innate immune activation. Once cytosolic double-stranded DNA becomes available—regardless of whether it originates from micronuclear rupture, nuclear envelope instability, or replication-derived fragments—cGAS synthesizes the second messenger 2′3′-cGAMP, which activates STING at the endoplasmic reticulum [7,8]. Activated STING subsequently traffics to the Golgi, where it recruits TBK1 and phosphorylates IRF3 to induce type I interferon (IFN-α/β) and pro-inflammatory cytokine transcription [4,5]. Transient cGAS–STING signaling promotes dendritic-cell maturation, antigen cross-presentation, and CD8^+^ T-cell priming, thereby coordinating innate sensing with adaptive antitumor immunity [10,73]. In contrast, chronic or dysregulated pathway activation shifts signaling toward NF-κB-driven inflammation, PD-L1 upregulation, and accumulation of myeloid-derived suppressor cells, ultimately fostering an immunosuppressive microenvironment [74]. Thus, the immunologic outcome is determined not only by the magnitude but also by the duration of cGAS–STING activation [75]. Mechanistically, DDR-targeting therapies modulate this axis in therapeutically meaningful ways. PARP inhibitors in BRCA-deficient tumors promote cGAS–STING-dependent CD8^+^ T-cell activation and antitumor control [6]. Similarly, ATR inhibition amplifies radiation-induced inflammatory transcription, supporting rational combinations of DDR inhibitors with immunotherapy [11]. Together, these findings illustrate how DDR perturbation can convert genomic instability into immunogenic stress, forming the mechanistic basis for synthetic-lethal and immune-stimulatory strategies in precision oncology.

### 3.2. Neoantigen Generation and Adaptive Immune Recognition

DDR deficiency plays a central role in shaping the immunogenic landscape of cancer by elevating TMB and driving the formation of neoantigens—novel peptides derived from somatic mutations that are recognized by the adaptive immune system. The accumulation of replication errors in tumors harboring MMR or HRR defects generates abundant nonsynonymous mutations, leading to the presentation of altered peptides by major histocompatibility complex (MHC) molecules and subsequent activation of cytotoxic T lymphocytes (CTLs) [28,44]. This mutational landscape underlies the pronounced responsiveness of microsatellite instability-high (MSI-H) and MMR-deficient cancers to immune checkpoint inhibitors such as pembrolizumab, leading to the first tissue-agnostic FDA approval based on a molecular biomarker rather than tumor origin [76].

However, recent evidence indicates that neoantigen quality—characterized by MHC binding affinity, peptide stability, clonality, and T-cell receptor recognition potential—is more predictive of immunotherapy outcomes than absolute TMB alone [77,78]. Clonal neoantigens, which are shared by the majority of tumor cells, tend to elicit stronger and more durable antitumor T-cell responses, whereas subclonal neoantigens are associated with reduced checkpoint blockade efficacy due to intratumoral heterogeneity and immune escape [79]. Moreover, tumors evolve dynamically under immune pressure through immunoediting, a Darwinian process that selects for variants capable of evading immune recognition. This occurs through the loss of highly immunogenic neoepitopes, defects in antigen-processing and presentation machinery (e.g., B2M, HLA mutations), or the upregulation of immune checkpoint ligands such as PD-L1 [80,81].

Thus, while DDR defects heighten the potential for immune visibility, the net immunologic outcome depends on selective pressures within the tumor microenvironment. Chronic exposure to immune surveillance promotes adaptive resistance, including antigen loss, T-cell exhaustion, and metabolic reprogramming of the tumor milieu, all of which can diminish the efficacy of immune checkpoint blockade [82]. Therefore, integrating genomic indicators of DDR dysfunction with neoantigen profiling and immune landscape features is essential for refining predictive biomarkers and optimizing personalized immunotherapy strategies [62].

### 3.3. Type I Interferon Signaling and Immune Modulation

The interferon (IFN) response triggered downstream of cGAS–STING activation represents a crucial axis connecting DNA damage to immune modulation. Upon sensing cytosolic DNA, the STING–TBK1–IRF3 cascade induces transcription of type I interferons (IFN-α/β) and numerous interferon-stimulated genes (ISGs) that shape both innate and adaptive immunity [5].

Acute activation of type I IFN signaling enhances antigen presentation by up-regulating MHC class I and promoting the acquisition of tumor-derived peptide-MHC complexes by dendritic cells. These changes facilitate CD8+ T-cell priming and contribute to anti-tumor immunity [83,84]. Transient exposure to type I interferons has been shown to promote the recruitment of Batf3^+^ dendritic cells and enhance CD8^+^ T-cell priming in the context of tumor-derived DNA damage, linking innate sensing of genotoxic stress to adaptive immune activation [85].

In contrast, chronic or dysregulated type I IFN signaling can promote immune evasion. Sustained activation of the STAT1–IRF1 axis induces inhibitory mediators such as PD-L1 and IDO1, contributing to an “IFN-adaptive” state that impairs T-cell effector functions and supports resistance to immune checkpoint blockade [86,87,88]. A sustained interferon-stimulated gene (ISG) program can promote tumor-intrinsic survival pathways, including enhanced DNA damage response and metabolic reprogramming, which together attenuate immunogenic cell death and contribute to reduced responsiveness to therapy. Moreover, prolonged IFN-I exposure drives progressive CD8^+^ T-cell exhaustion, characterized by sustained expression of inhibitory receptors (PD-1, LAG-3, TIM-3) and diminished cytotoxic capacity, further reinforcing the immunosuppressive consequences of chronic IFN signaling [89].

The balance between beneficial and maladaptive IFN-I signaling is increasingly recognized as a determinant of therapeutic outcome. Short-term, high-amplitude IFN-I pulses promote tumor rejection, whereas prolonged low-level signaling drives CD8^+^ T cell exhaustion. Experimental modulation of STING or downstream transcription factors has shown that controlling the duration and intensity of pathway activation can restore responsiveness to immune checkpoint inhibitors in otherwise refractory tumors [90,91,92].

Taken together, these data highlight that the amplitude and temporal dynamics of DDR-driven type I interferon signaling critically dictate whether the outcome will be immune stimulation or immune suppression. Therapeutic strategies that fine-tune IFN-I activity—by combining DDR inhibitors, STING agonists, or IFN-pathway modulators—offer a promising route to enhance antitumor immunity while avoiding chronic interferon-induced resistance.

### 3.4. Immune Checkpoint Regulation and Tumor Evasion

Across diverse tumor types, DDR dysfunction is closely linked to the transcriptional induction of immune checkpoints that restrain cytotoxic activity within the tumor microenvironment. Genotoxic stress and unrepaired DNA lesions activate the cGAS–STING pathway, leading to type I interferon production and subsequent JAK–STAT signaling; this cytokine-driven activation of STAT1/STAT3 and IRF1 is the primary mechanism that induces CD274 (PD-L1) expression, rather than direct signaling through ATM, ATR, or CHK1 [93,94]. This pathway has been confirmed in both genotoxic stress and radiotherapy models, where activation of ATM/ATR signaling increases PD-L1 expression via IRF1, contributing to attenuation of IFN-driven T-cell responses and promoting short-term survival of stressed tumor cells [95]. Such DDR-dependent checkpoint induction represents a tumor-intrinsic adaptation that simultaneously prevents immune overactivation and facilitates immune evasion under chronic DNA damage conditions [71].

Paradoxically, these same mechanisms can create therapeutic vulnerabilities. Tumors with HRR deficiency, including BRCA1/2 mutations, often display elevated PD-L1 expression and an inflamed transcriptomic profile, rendering them more susceptible to PD-1/PD-L1 blockade. Retrospective and mechanistic studies have shown that such tumors exhibit higher interferon signatures and immune checkpoint expression, correlating with improved responses to immune checkpoint inhibitors (ICIs) [96,97].

Preclinical and early-phase clinical data strongly support synergistic efficacy between PARP inhibition and checkpoint blockade. PARP inhibitors trigger accumulation of cytosolic DNA fragments that activate cGAS–STING signaling, enhance dendritic-cell recruitment, and promote CD8^+^ T-cell infiltration. Combination treatment with PARP inhibitors (e.g., olaparib or niraparib) and anti-PD-L1 therapy has demonstrated superior tumor regression in BRCA1/2-mutant models compared with either monotherapy [98,99].

Preclinical and early-phase clinical data strongly support the synergistic efficacy of combining PARP inhibition with immune checkpoint blockade. PARP inhibitors such as olaparib and niraparib promote the accumulation of cytosolic DNA fragments that activate cGAS–STING signaling, enhance dendritic-cell recruitment, and increase CD8^+^ T-cell infiltration. In BRCA1/2-mutant tumor models, this combination has demonstrated superior tumor regression compared to either monotherapy [98,100]. Translational results from the MEDIOLA and TOPACIO phase II trials further demonstrate manageable safety profiles and durable responses with PARP + PD-L1/PD-1 blockade combinations in ovarian, breast, and prostate cancers [101].

Beyond PARP inhibition, new DDR-targeting strategies—including ATR, DNA-PK, and WEE1 inhibitors—are being tested in combination with ICIs to potentiate immunogenic DNA damage while minimizing systemic toxicity. Early-phase studies of elimusertib (BAY 1895344), peposertib (M3814), and adavosertib (AZD1775) combined with anti–PD-1/PD-L1 antibodies have shown preliminary antitumor activity in solid tumors with DDR mutations or replication-stress signatures [102,103]. These combinatorial approaches aim to reprogram DDR-driven immune suppression into therapeutic vulnerability by converting genotoxic stress into immunogenic cell death and durable checkpoint sensitivity.

### 3.5. The Bidirectional Feedback Loop Between DDR and Immunity

The relationship between the DDR and immune signaling operates as a reciprocal feedback system, in which genotoxic stress promotes immune activation while immune effector mechanisms further propagate DNA damage [104]. This dynamic interplay influences whether immune surveillance leads to tumor elimination or fosters an immunosuppressive equilibrium within the tumor microenvironment [105].

DNA damage stimulates immune activation through multiple convergent mechanisms. Defective repair of DSBs and replication stress leads to the accumulation of cytosolic DNA fragments that activate the cGAS–STING pathway, triggering type I interferon secretion and enhancing dendritic-cell recruitment and T-cell priming [61]. In parallel, DDR deficiency increases TMB and neoantigen load, broadening the repertoire of epitopes recognizable by cytotoxic lymphocytes. Collectively, these mechanisms transform genomic instability into immunogenic stress, a process that enhances the efficacy of immune checkpoint blockade when appropriately regulated [71].

Conversely, immune effector molecules themselves can induce or amplify DNA damage, reinforcing DDR activation. Cytotoxic T lymphocytes (CTLs) and natural killer (NK) cells release granzyme B and perforin, which trigger apoptotic DNA fragmentation in tumor targets. This process generates secondary double-strand breaks (DSBs) and micronuclei that sustain cGAS–STING signaling, creating a self-reinforcing loop between immune attack and genomic instability [106]. Additionally, macrophage- and T-cell-derived reactive oxygen species (ROS) and nitric oxide (NO) impose oxidative DNA lesions, leading to activation of ATM/ATR kinases and transcriptional induction of DNA repair genes that modulate immune outcomes [107,108].

This bidirectional signaling loop establishes a regulatory balance between immunostimulation and immunosuppression. Acute activation of the DDR enhances tumor immunogenicity by promoting antigen release, dendritic-cell priming, and cytotoxic T-cell infiltration [71]. Conversely, chronic DNA damage and sustained interferon signaling can lead to immunoediting, upregulation of immune checkpoints, and exhaustion of effector lymphocytes, ultimately favoring tumor persistence [64,109]. The equilibrium between these forces determines whether DDR–immune crosstalk results in tumor eradication or tolerance under therapy.

Understanding and therapeutically manipulating this feedback axis holds significant translational value. Controlled induction of immunogenic DNA damage through PARP, ATR, or DNA-PK inhibition—combined with immune checkpoint blockade or STING agonism—has been proposed as a strategy to shift the balance toward antitumor immunity while minimizing the deleterious effects of chronic inflammation [94,100,110]. Emerging preclinical and early clinical data suggest that such combinations may enhance immune activation, although their full therapeutic potential and optimal implementation remain to be defined. These insights reinforce that the DDR–immunity circuit is not a linear pathway but a dynamic, self-sustaining feedback network whose fine-tuning may ultimately determine therapeutic benefit in DDR-targeted and immunomodulatory settings.

## 4. Therapeutic Targeting of DDR–Immune Crosstalk: Clinical Advances and Challenges

The growing understanding of how DDR pathways intersect with immune signaling has opened new avenues for therapeutic innovation [71]. Modern oncology increasingly leverages these interactions to enhance tumor immunogenicity, overcome resistance mechanisms, and achieve durable clinical responses. The current therapeutic landscape integrates DDR inhibitors, immune checkpoint blockade, and innate immune modulators as the foundation of next-generation combinatorial strategies [111].

### 4.1. DDR Inhibition as an Immunogenic Strategy

Pharmacologic inhibition of DDR pathways has emerged not only as a strategy to induce synthetic lethality in repair-deficient tumors but also as a potent driver of tumor immunogenic reprogramming. By promoting the accumulation of unrepaired DNA breaks and replication intermediates, DDR inhibitors generate cytosolic DNA that activates the cGAS–STING–TBK1–IRF3 axis, leading to type IFN-I production and recruitment of immune effector cells [112,113]. This immunogenic stress converts intrinsic DNA damage into a “danger signal,” effectively bridging genotoxic stress with innate and adaptive antitumor immunity.

Among DDR-targeting agents, poly(ADP-ribose) polymerase (PARP) inhibitors—such as olaparib, niraparib, and talazoparib—are the most clinically validated. Beyond their canonical role in trapping PARP1 at DNA single-strand breaks, these agents also elicit STING-dependent interferon responses in homologous recombination–deficient tumors [98]. This immunogenic effect promotes dendritic-cell activation and cytotoxic T-cell infiltration, establishing a secondary immune mechanism that complements their direct cytotoxicity [114]. In addition, PARP inhibition has been shown to upregulate antigen presentation machinery—including MHC class I and TAP1/2—and increase chemokines such as CXCL10 and CCL5, thereby fostering a tumor microenvironment permissive to T-cell recruitment [115,116].

This adaptive immune feedback loop, while enhancing immune recognition, also triggers compensatory PD-L1 upregulation on tumor cells via STAT1–IRF1 signaling. This response can be therapeutically exploited through rational combination with immune checkpoint blockade [117]. Multiple preclinical models have demonstrated robust synergy between PARP inhibitors and PD-1/PD-L1 antagonists, resulting in durable tumor regression and expansion of CD8^+^ T-cell repertoires [117,118,119,120].

Early-phase clinical studies—including MEDIOLA, TOPACIO, and KEYLYNK-007—have confirmed the feasibility and safety of combining PARP inhibitors with immune checkpoint blockade, particularly in BRCA1/2-mutated breast, ovarian, and prostate cancers [101,121]. Beyond PARP inhibition, ATR inhibitors—such as ceralasertib and elimusertib—act by inducing replication fork collapse and accumulation of double-strand breaks during S-phase, which activates innate immune pathways and enhances tumor sensitivity to both radiotherapy and immunotherapy [122,123]. ATR inhibition amplifies cytosolic DNA accumulation and upregulates inflammatory chemokines such as CXCL10 via STING-dependent mechanisms, thereby potentiating antitumor T-cell recruitment [124,125].

Recent clinical trials—including NCT03772561 and NCT04266912—are evaluating ATR inhibitors in combination with PD-1 or PD-L1 antibodies, aiming to leverage immunogenic replication stress while mitigating systemic toxicity [126,127]. Overall, DDR inhibition functions as a two-pronged immunogenic strategy—directly inducing tumor cell death while simultaneously converting genomic instability into a proinflammatory, interferon-rich state that can be harnessed by checkpoint blockade or STING agonism. Optimizing this interplay requires precise temporal control of DDR-targeting therapies to maximize immune activation without provoking chronic inflammatory resistance.

In parallel with these mechanistic advances, targeted delivery platforms are being developed to improve the therapeutic index of DDR inhibitors. Nanoparticle-based formulations of PARP and ATR inhibitors, including lipid nanoparticles, polymeric micelles, and albumin-bound conjugates, can increase intratumoral drug accumulation while limiting off-target hematologic toxicity. Ligand-directed carriers and antibody–drug conjugates that exploit overexpressed surface receptors on DDR-defective tumors further refine spatial selectivity, whereas pH-, redox-, or enzyme-responsive prodrugs enable controlled release within the tumor microenvironment. These delivery innovations, highlighted in recent translational oncology and cancer progress reports, are expected to widen the window in which DDR-targeting agents can be safely combined with radiotherapy and immune checkpoint blockade [128].

### 4.2. Combination Strategies with Immune Checkpoint Blockade

ICIs targeting PD-1, PD-L1, and CTLA-4 have significantly transformed cancer therapy, especially in lung and colorectal cancers. However, many patients exhibit primary resistance or develop acquired resistance over time. Recent studies suggest that combining ICIs with DDR inhibitors may enhance therapeutic efficacy by increasing tumor immunogenicity and promoting sustained immune activation [129,130].

Mechanistically, inhibition of DDR pathways enhances tumor immunogenicity by increasing mutational burden, promoting cytosolic DNA accumulation, and activating type I interferon signaling. These events generate a pro-inflammatory tumor microenvironment that facilitates T-cell infiltration and improves responsiveness to immune checkpoint blockade [94].

PARP inhibitors are among the most extensively investigated agents in combination with ICIs. In BRCA1/2-mutant breast, ovarian, and prostate cancers, PARP blockade combined with anti-PD-1/PD-L1 antibodies—such as durvalumab and pembrolizumab—has shown improved response rates and prolonged progression-free survival compared to monotherapy. Phase II trials like MEDIOLA (olaparib + durvalumab) and TOPACIO/KEYNOTE-162 (niraparib + pembrolizumab) demonstrated manageable safety profiles and durable responses, particularly in patients with homologous recombination deficiency (HRD) [131,132].

In metastatic castration-resistant prostate cancer (mCRPC), the phase III KEYLYNK-010 trial evaluated the combination of olaparib and pembrolizumab versus next-generation hormonal agents. Although the study was terminated early due to lack of overall clinical benefit, exploratory subgroup analyses suggested potential antitumor activity in patients harboring DDR gene alterations [133,134,135].

ATR inhibitors such as ceralasertib (AZD6738) and elimusertib (BAY 1895344) are currently under active investigation in combination with immune checkpoint inhibitors (ICIs). Mechanistically, ATR inhibition induces replication-fork collapse and activates innate immune pathways, including STING-mediated chemokine signaling, which promotes intratumoral CD8^+^ T-cell infiltration [122]. Early-phase clinical trials and preclinical models have reported encouraging immune responses, particularly in tumors with ATM deficiency or HRR impairment [123]. A similar rationale supports emerging studies investigating the combination of DNA-PK and WEE1 inhibitors with ICIs. These strategies aim to amplify immunogenic DNA damage—through replication stress and impaired repair—while maintaining manageable toxicity [71]. Preclinical and early-phase clinical data suggest that targeting these DDR kinases may enhance antitumor immunity, particularly in tumors with defective cell cycle checkpoints or DNA repair pathways [88].

Optimizing treatment scheduling and dose intensity is critical in DDR-ICI combinations. Excessive or chronic DNA damage may trigger maladaptive type I interferon signaling, leading to PD-L1 overexpression and recruitment of myeloid-derived suppressor cells (MDSCs), ultimately dampening antitumor immune responses and promoting immune evasion [93,136,137]. Sequential or intermittent administration of DDR inhibitors in combination with ICIs has been shown to mitigate immune exhaustion and preserve antitumor immunity. This approach helps avoid maladaptive interferon signaling and excessive PD-L1 induction, maintaining a more favorable tumor microenvironment for immune activation [71].

Altogether, combination strategies integrating DDR inhibition with immune checkpoint blockade exemplify the paradigm of immunogenic precision oncology—leveraging replication stress and genomic instability to expand the therapeutic window of immunotherapy.

### 4.3. STING Agonists and Innate Immune Modulation

Beyond immune-checkpoint blockade, pharmacologic activation of the stimulator of interferon genes (STING) pathway has emerged as a promising strategy to mimic the immunogenic effects of DNA damage-induced cytosolic DNA sensing [138]. Synthetic cyclic dinucleotide (CDN) ligands such as 2′3′-cGAMP and next-generation small-molecule STING agonists directly activate TBK1–IRF3 signaling, leading to rapid type I interferon release, dendritic-cell maturation, and cross-priming of CD8^+^ T cells. This innate immune activation transforms “cold,” non-inflamed tumors into T-cell-permissive environments that can respond to immune checkpoint inhibition [139,140].

Early clinical translation of STING agonists has been constrained by pharmacokinetic limitations and systemic toxicity. First-generation cyclic dinucleotides (CDNs) such as ADU-S100 (MIW815) [141] and MK-1454 [142] demonstrated potent local interferon induction but suffered from poor bioavailability and dose-limiting inflammation in phase I trials. To address these challenges, nanoparticle-encapsulated and tumor-targeted STING agonists have been developed to localize signaling within the tumor microenvironment [143,144]. These delivery platforms enable sustained intratumoral activation with reduced systemic exposure, representing a critical step toward broader clinical applicability.

Rational combination strategies have demonstrated that STING agonists synergize with DNA-damage-inducing therapies such as PARP and ATR inhibitors [104]. Preclinical studies show that this pairing enhances type I interferon transcription, promotes dendritic-cell recruitment, and drives robust infiltration of cytotoxic CD8^+^ T cells—achieving immune activation levels beyond those induced by either agent alone [101,140].

Collectively, these findings position STING agonists as pharmacologic mimics of DNA damage response (DDR)-driven immunogenicity. By integrating optimized delivery platforms with DDR inhibitors and immune checkpoint blockade, next-generation triplet regimens aim to harness innate immune activation while mitigating chronic inflammation and T-cell exhaustion associated with sustained interferon signaling. Ongoing phase I/II trials are currently evaluating these strategies to establish safety profiles and durable antitumor responses in solid tumors.

### 4.4. Emerging Therapeutic Agents and Biomarker Integration

The therapeutic landscape at the DDR–immune interface is rapidly evolving. Novel inhibitors targeting WEE1, CHK1, DNA-PKcs, and POLθ are expanding beyond PARP and ATR blockade, intensifying replication stress and DNA damage to elicit innate immune activation [39]. These agents sensitize tumors to immunotherapy, and ongoing clinical trials are now combining them with immune checkpoint blockade, adoptive T-cell transfer, or oncolytic virotherapy to broaden therapeutic responsiveness across diverse tumor types [94,145].

In parallel, the integration of predictive biomarkers is becoming central to tailoring DDR–immune combination therapies. HRD scores, TMB, and STING pathway activation signatures are actively being explored as indicators of therapeutic responsiveness [71]. These biomarkers help identify patients most likely to benefit from DNA damage response inhibitors combined with immune checkpoint blockade or innate immune activators [146].

Emerging studies highlight the value of composite biomarkers that integrate genomic instability indices with transcriptomic immune profiles to refine patient selection and balance therapeutic efficacy with immune-related toxicity [71,147]. These advances signal a transition toward precision immunogenomics, where DDR-targeted agents are deployed based on molecular signatures that predict both clinical benefit and immunologic risk.

In addition to these composite signatures, several translational biomarkers are gaining traction for clinical decision-making. Functional HRD assays, γH2AX or RAD51 foci quantification, and replication stress signatures have shown predictive value for selecting patients most likely to respond to PARP or ATR inhibition [148]. Complementarily, immune-related biomarkers such as interferon-stimulated gene (ISG) scores, T-cell inflamed GEP signatures, and spatial patterns of CD8^+^ infiltration help identify tumors with pre-existing inflammatory activity that may synergize with DDR–ICI combination therapies [149]. Adaptive resistance markers—including chronic STING activation profiles, upregulation of PD-L1 and TIM-3, or defects in antigen-presentation machinery—are increasingly recognized as determinants of treatment failure and may guide the development of next-generation triplet strategies involving DDR inhibitors, ICI, and innate immune modulators [148].

To illustrate the translational relevance of DDR–immune targeting strategies, Table 2 summarizes selected clinical trials that have explored or are currently investigating combination therapies integrating DNA repair inhibition with immunomodulatory agents. These studies demonstrate the therapeutic potential of exploiting genomic instability to enhance antitumor immunity, while also revealing the diversity of trial designs, targeted mechanisms, and clinical outcomes. Collectively, they provide a description of ongoing efforts to refine patient selection and optimize synergistic interactions within the DDR–immune therapeutic axis.

### 4.5. Translational and Clinical Challenges

Despite encouraging progress, translating DDR–immune combination therapies into durable clinical benefit remains challenging. Tumor heterogeneity leads to variable dependency on DNA damage response pathways and divergent immune responsiveness, highlighting the need for individualized therapeutic strategies guided by molecular and immunologic profiling [71,150].

Achieving an optimal therapeutic window remains a major challenge in DDR–immune combination therapies. Excessive DDR inhibition can lead to hematologic toxicity, chronic interferon signaling, and myelosuppression, while insufficient inhibition fails to generate immunogenic stress. Additionally, adaptive resistance may arise through restoration of homologous recombination repair, attenuation of STING pathway signaling, or compensatory upregulation of alternative immune checkpoints [71,149].

To overcome current limitations, future research should integrate spatial transcriptomics, single-cell multiomics, and machine-learning-based modeling to map DDR–immune interactions at cellular resolution and predict therapeutic outcomes. Incorporating longitudinal immune monitoring into clinical trials will clarify how DDR-targeted therapies reshape the immune landscape over time, enabling more precise and safer combination strategies [151,152].

## 5. Heterogeneity of DDR Defects and Their Impact on Immune Crosstalk and Therapeutic Response

DDR alterations exhibit substantial heterogeneity across cancer types and within individual tumors, leading to distinct immunologic and therapeutic outcomes. Even within homologous recombination–deficient cancers, BRCA1 and BRCA2 loss generate non-identical phenotypes: BRCA1 deficiency is strongly associated with replication stress, cytosolic DNA accumulation, and robust cGAS–STING activation, whereas BRCA2 loss produces fewer cytosolic DNA fragments and a weaker inflammatory response due to its more specific role in RAD51 loading [30]. In contrast, loss of upstream kinases such as ATM or ATR shapes immunity through different mechanisms—ATM-deficient tumors display high genomic instability and enhanced type I interferon signaling, whereas ATR loss compromises replication-fork stability and shifts immune activation toward replication-catastrophe–driven inflammatory signaling [153].

Recent single-cell and spatial transcriptomic studies further demonstrate marked intratumoral variability in DDR activity, with coexisting subclones displaying divergent levels of replication stress, micronuclei formation, and cGAS–STING signaling. This heterogeneity profoundly influences therapeutic sensitivity: PARP and ATR inhibitors preferentially target subclones with high replication stress and active STING signaling, while neighboring DDR-proficient or STING-silent regions may persist and drive therapeutic resistance [139,154]. Similarly, cancer-type-specific DDR landscapes—such as the strong STING activation signature in triple-negative breast cancers versus the more muted response in prostate and ovarian tumors—modulate responsiveness to immunotherapy and shape rational DDR–IO combinations [155].

Taken together, these observations indicate that DDR–immune crosstalk is not uniform but instead highly dependent on the specific gene defect, tumor lineage, and intratumoral context. Accounting for this heterogeneity is essential for predicting therapeutic response and designing effective precision strategies integrating PARP inhibition, ATR blockade, and immunotherapy.

## 6. Temporal Dynamics of DDR Inhibition and Implications for Sequencing with Immunotherapy

The immunological consequences of DDR inhibition are highly dependent on timing, dose, and treatment sequence. Acute DDR blockade—such as short-term PARP or ATR inhibition—induces rapid accumulation of cytosolic DNA, robust cGAS–STING activation, type I interferon release, and increased antigen presentation, thereby creating a transient window of heightened tumor immunogenicity [6]. In contrast, chronic or continuous DDR inhibition can lead to STING desensitization, sustained NF-κB activation, and persistent PD-L1 upregulation, ultimately promoting an immunosuppressive microenvironment. These opposing outcomes highlight the importance of optimizing treatment schedules to preserve pro-inflammatory signaling while avoiding chronic inhibitory states [119].

Several preclinical studies demonstrate that sequential rather than concurrent administration of DDR inhibitors with immune checkpoint inhibitors (ICIs) produces superior antitumor immunity. Short-term PARP or ATR inhibition prior to PD-1/PD-L1 blockade enhances dendritic-cell priming and CD8^+^ T-cell infiltration, whereas simultaneous administration may blunt interferon signaling due to early checkpoint activation or STING exhaustion. DNA-PKcs inhibition similarly shows timing-dependent effects, with intermittent dosing preserving STING responsiveness and potentiating anti-PD-1 therapy more effectively than continuous exposure [6,77,119].

Emerging clinical data mirror these findings. In trials combining PARP inhibitors with pembrolizumab or durvalumab, treatment schedules incorporating an induction phase of PARP inhibition before ICI initiation showed more durable responses than fully concurrent regimens [6,156,157]. Early-phase ATR inhibitor trials have likewise suggested that brief priming doses administered 24–72 h prior to immunotherapy maximize IFN-β induction and T-cell recruitment [158]. Together, these studies indicate that rational sequencing—particularly short-pulse DDR inhibition followed by checkpoint blockade—may optimize therapeutic synergy while limiting immunosuppressive adaptation [159].

Collectively, these observations underscore the need to incorporate temporal dynamics into the design of DDR–immunotherapy combinations. The balance between acute immunostimulatory signaling and chronic immunosuppressive responses should guide decisions regarding dose intensity, dosing interval, and sequential versus concurrent administration in future clinical trials.

## 7. Spatial and Single-Cell Insights into DDR–Immune Crosstalk

Recent advances in spatial transcriptomics and single-cell sequencing have revealed that DDR signaling, cytosolic DNA accumulation, and immune activation are far more heterogeneous than suggested by bulk-level analyses [151,160]. High-resolution studies demonstrate that micronuclei formation and cGAS–STING activation occur in spatially restricted tumor regions enriched for replication stress and chromosomal instability [161]. For example, spatial profiling by Jin et al. (2024) identified micronuclei “hotspots” within proliferative tumor zones, where focal STING activation coincided with localized type I interferon transcription and dendritic-cell recruitment. In contrast, adjacent tumor areas showed minimal STING pathway activity despite comparable genomic alterations, underscoring the spatial compartmentalization of DDR-driven immune signaling [162].

Single-cell atlases from Li et al. (2024) and Wang et al. (2021) further reveal striking subclonal diversity in DDR pathway integrity. Subpopulations with BRCA1/2 loss display strong interferon signatures and enhanced antigen presentation, whereas neighboring subclones harboring reversion mutations or partial HRR restoration exhibit STING-silent phenotypes and impaired immunogenicity [163,164]. These subclonal differences strongly influence therapeutic responses: PARP inhibitors selectively eliminate STING-active, replication-stressed subclones, but spare DDR-restored populations that subsequently drive resistance and immune evasion.

Spatial multiomics studies in breast, ovarian, and prostate cancers confirm that immune infiltration mirrors the regional architecture of DDR activation [165]. CD8^+^ T cells preferentially accumulate in STING-high regions, while immunosuppressive macrophages and myeloid-derived suppressor cells dominate STING-low niches [166]. These spatially segregated ecosystems explain why DDR–IO combinations produce heterogeneous intratumoral responses and why immune reinvigoration often remains incomplete despite systemic drug exposure.

Together, these findings indicate that DDR–immune crosstalk is not uniform throughout tumors but instead emerges from localized patterns of replication stress, cytosolic DNA release, and pathway reversion events. Incorporating spatial and single-cell data into therapeutic design will be essential for predicting which tumor regions—and which subclones—are likely to respond to PARP or ATR inhibition, and for identifying combination strategies that overcome spatially restricted immune escape.

## 8. Conclusions and Future Perspectives

The expanding understanding of DDR mechanisms has reshaped cancer biology, revealing that DDR not only safeguards genomic integrity but also plays a pivotal role in modulating antitumor immunity. Deficiencies in DNA repair can simultaneously enhance tumor immunogenicity and promote immune evasion, highlighting the complex and bidirectional nature of DDR–immune interactions.

This duality presents unique therapeutic opportunities. By targeting DDR pathways, it is possible to reprogram the tumor microenvironment and sensitize otherwise resistant cancers to immunotherapy. Clinical success with PARP and ATR inhibitors has demonstrated that genotoxic stress can amplify immune activation—provided that treatment intensity and timing are carefully optimized to avoid immune exhaustion and systemic toxicity.

To translate these insights into durable clinical benefit, future strategies must address tumor heterogeneity, which drives variable dependency on DDR pathways and divergent immune responsiveness. Precision approaches will require robust biomarkers—such as HRD scores, TMB, and STING activation signatures—as well as composite profiles that integrate genomic instability with immune transcriptomics.

Advanced technologies like spatial transcriptomics, single-cell multiomics, and machine-learning-based modeling will be essential to map DDR–immune interactions at cellular resolution. Incorporating longitudinal immune monitoring into clinical trials will further clarify how DDR-targeted therapies reshape the immune landscape over time and guide rational combination strategies.

Looking ahead, the therapeutic paradigm is shifting toward precision immunogenomics, where DDR-targeted agents are deployed based on molecular signatures that predict both efficacy and immunologic risk. Combinations involving STING agonists, DDR inhibitors, and immune checkpoint blockade hold promise for converting immunologically “cold” tumors into responsive phenotypes. Continued translational research and adaptive trial designs will be key to safely harnessing these mechanisms.

Ultimately, targeting the DDR–immune interface represents a transformative frontier in oncology—bridging genomic instability with immune control and paving the way for more personalized, durable, and mechanism-driven cancer therapies.

## Figures and Tables

**Table 1:** Main DNA damage response (DDR) pathways, their key molecular components, immunologic consequences, and therapeutic implications in cancer.

DDR Pathway	Key Molecular Components	Primary Immunologic Consequence	Representative Therapeutic Implication
Base Excision Repair (BER) [1,2,3]	PARP1, XRCC1, DNA polymerase β	Cytosolic DNA accumulation and cGAS–STING activation upon PARP inhibition	PARP inhibitors (olaparib, niraparib) enhance immunogenic cell death in HR-deficient tumors
Nucleotide Excision Repair (NER) [4,5]	XPC, ERCC1, XPF, XPG	Repair of UV-induced bulky adducts; limited direct immune activation	Synergy with immune checkpoint inhibitors in UV-associated cancers (e.g., melanoma)
Mismatch Repair (MMR) [6,7,8]	MLH1, MSH2, MSH6, PMS2	Microsatellite instability (MSI), increased mutational load and neoantigen formation	Predictive biomarker for PD-1/PD-L1 blockade sensitivity
Homologous Recombination Repair (HRR) [9,10,11]	BRCA1, BRCA2, RAD51	Cytosolic DNA leakage, type I IFN response via cGAS–STING activation	PARP and ATR inhibitors combined with immunotherapy
Non-Homologous End Joining (NHEJ) [12,13]	KU70/80, DNA-PKcs, XRCC4	Chromosomal instability; limited direct immunogenic signaling	DNA-PK inhibitors investigated for radiosensitization and immune modulation

**Table 2:** Representative clinical trials exploring combination therapies that integrate DNA damage response (DDR) inhibition with immune checkpoint blockade or innate immune modulation.

Combination Strategy	Mechanistic Rationale	Cancer Type	Clinical Phase	Key Observations/Outcome
Olaparib + Durvalumab (anti–PD-L1) [118,119,120,121]	PARP inhibition increases cytosolic DNA and PD-L1 expression via STING activation	Ovarian, breast (BRCA1/2-mutant)	II	Improved response rate and immune infiltration; manageable toxicity
Niraparib + Dostarlimab (anti–PD-1) [122,123,124,125]	Synthetic lethality + checkpoint blockade enhances neoantigen-driven immunity	Endometrial (HRD^+^)	III	Significant PFS benefit in HRD-positive subgroup
Ceralasertib (ATR inhibitor) + Pembrolizumab (anti–PD-1) [126,127,128,129,130]	Replication stress–induced DNA damage activates STING and enhances T-cell recruitment	NSCLC, melanoma	II	Enhanced CD8^+^ infiltration, delayed progression
Talazoparib + Avelumab (anti–PD-L1) [131,132,133,134,135]	PARP inhibition potentiates immunotherapy through cGAS–STING activation	Prostate, breast	II	Promising disease control rate; ongoing evaluation
STING agonist (ADU-S100) + Checkpoint blockade [88,136,137,138,139]	Pharmacologic activation of innate immune sensing mimics DDR deficiency	Solid tumors	I/II	Early immune activation; variable durability of response
DNA-PK inhibitor (M3814) + Radiotherapy + ICI [140,141,142,143,144,145]	DNA-PK blockade amplifies immunogenic cell death following genotoxic stress	Head and neck, NSCLC	I/II	Enhanced T-cell infiltration; safety profile under investigation

## Data Availability

No new data were created or analyzed in this study. Data sharing is not applicable to this article.

## Abbreviations

The following abbreviations are used in this manuscript:

ATM	Ataxia–telangiectasia mutated
ATR	ATM and Rad3-related
BER	Base Excision Repair
cGAMP	Cyclic GMP–AMP
cGAS	Cyclic GMP–AMP Synthase
CHK1	Checkpoint Kinase 1
CHK2	Checkpoint Kinase 2
CIN	Chromosomal Instability
DC	Dendritic Cell
DDR	DNA Damage Response
DNA-PKcs	DNA-dependent Protein Kinase Catalytic Subunit
DSB	Double-Strand Break
HRR	Homologous Recombination Repair
HRD	Homologous Recombination Deficiency
ICI	Immune Checkpoint Inhibitor
ICD	Immunogenic Cell Death
IFN	Interferon
IFN-I	Type I Interferons
IR	Ionizing Radiation
IRF3	Interferon Regulatory Factor 3
ISG	Interferon-Stimulated Gene
JAK	Janus Kinase
KU70/80	Ku Heterodimer (XRCC6/XRCC5)
MDSC	Myeloid-Derived Suppressor Cell
MHC	Major Histocompatibility Complex
MMR	Mismatch Repair
MSI	Microsatellite Instability
MRN	MRE11–RAD50–NBS1 Complex
NER	Nucleotide Excision Repair
NF-κB	Nuclear Factor kappa-light-chain-enhancer of activated B cells
NSCLC	Non–Small Cell Lung Cancer
PARP	Poly(ADP-Ribose) Polymerase
PD-1	Programmed Cell Death Protein 1
PD-L1	Programmed Death-Ligand 1
PIKKs	Phosphatidylinositol 3-Kinase-Related Kinases
RPA	Replication Protein A
SSB	Single-Strand Break
STING	Stimulator of Interferon Genes
TBK1	TANK-Binding Kinase 1
TME	Tumor Microenvironment
UV	Ultraviolet Radiation
XRCC1	X-Ray Repair Cross-Complementing Protein 1
